# Adjuvant low-frequency rTMS in treating auditory hallucinations in recent-onset schizophrenia: a randomized controlled study investigating the effect of high-frequency priming stimulation

**DOI:** 10.1186/s12991-015-0046-2

**Published:** 2015-02-15

**Authors:** Prasenjit Ray, Vinod Kumar Sinha, Sai Krishna Tikka

**Affiliations:** Department of Psychiatry, Burdwan Medical College, Burdwan, 713101 West Bengal India; KS Mani Center for Cognitive Neurosciences and Department of Psychiatry, Central Institute of Psychiatry, Kanke, Ranchi, 834006 Jharkhand India

**Keywords:** Schizophrenia, Priming TMS, Transcranial magnetic stimulation, Auditory verbal hallucinations, Randomized controlled trial, Recent onset

## Abstract

**Background:**

Repetitive transcranial magnetic stimulation (rTMS) has been found to be effective in reducing frequency and duration of auditory verbal hallucinations (AVH). Priming stimulation, which involves high-frequency rTMS stimulation followed by low-frequency rTMS, has been shown to markedly enhance the neural response to the low-frequency stimulation train. However, this technique has not been investigated in recent onset schizophrenia patients. The aim of this randomized controlled study was to investigate whether the effects of rTMS on AVH can be enhanced with priming rTMS in recent onset schizophrenia patients.

**Methods:**

Forty recent onset schizophrenia patients completed the study. Patients were randomized over two groups: one receiving low-frequency rTMS preceded by priming and another receiving low-frequency rTMS without priming. Both treatments were directed at the left temporo-parietal region. The severity of AVH and other psychotic symptoms were assessed with the auditory hallucination subscale (AHRS) of the Psychotic Symptom Rating Scales (PSYRATS), the Positive and Negative Syndrome Scale (PANSS) and the Clinical Global Impression (CGI).

**Results:**

We found that all the scores of these ratings significantly reduced over time (i.e. baseline through 1, 2, 4 and 6 weeks) in both the treatment groups. We found no difference between the two groups on all measures, except for significantly greater improvement on loudness of AVH in the group with priming stimulation during the follow-ups (F = 2.72; p < .05).

**Conclusions:**

We conclude that low-frequency rTMS alone and high-frequency priming of low-frequency rTMS do not elicit significant differences in treatment of overall psychopathology, particularly AVH when given in recent onset schizophrenia patients. Add on priming however, seems to be particularly better in faster reduction in loudness of AVH.

## Introduction

Auditory verbal hallucinations (AVH) are among the commonly presenting positive symptoms in patients of schizophrenia. While more than 60% of patients with schizophrenia present with AVH [1], majority of them respond usually to antipsychotic regimens. However, about 25% to 30% of patients who experience AVH have been found to be unresponsive to antipsychotic medication [2]. Among the non pharmacological strategies, psychological therapies have been found to be effective in reducing their burden but not able to cause much dent in frequency or duration [3].

Brain stimulation techniques, especially repetitive transcranial magnetic stimulation (rTMS) have been found to be effective in reducing frequency and duration of AVH. While both low and high-frequency (including theta burst), left and right temporo-parietal sites have been used as paradigms, low-frequency stimulation of left hemisphere has been found to be common as well as comparatively effective [4].

Less commonly used novel paradigms include priming rTMS, where low-frequency rTMS is preceded by a brief period of high-frequency rTMS. Priming stimulation, which involves high-frequency (6 Hz) rTMS stimulation followed by low-frequency rTMS (1Hz), has been shown to markedly enhance the neural response to the low-frequency stimulation train [5] and to have greater antidepressant effects than low-frequency rTMS alone when applied to right dorsolateral prefrontal cortex (DLPFC) [6,7]. As schizophrenia is associated with cortical hyperexcitability and deficits in cortical inhibition, it has been suggested that enhanced inhibition with priming stimulation would be more effective for refractory AVH and better tolerated than higher frequency stimulation paradigms [8,9]. Moreover, modulation of neural activity in the brain areas involved in the monitoring of inner speech has been proposed as a probable explanation for the effect of rTMS, particularly high frequency, on AVH [10].

Two randomized sham controlled studies, so far, have investigated this technique and found that it was not significantly better than 1-Hz rTMS [9,11]. Both these studies investigated patients with medication-resistant auditory hallucinations. Understanding the need for exploring the effects of rTMS in patients during the initial phases of psychosis, we conducting this priming rTMS experiment on recent onset patients with schizophrenia experiencing AVH with the hypothesis that priming stimulation would enhance the inhibitory action of low-frequency stimulation and would be better than sham priming.

## Methods and materials

### Subjects

**Figure 1:**
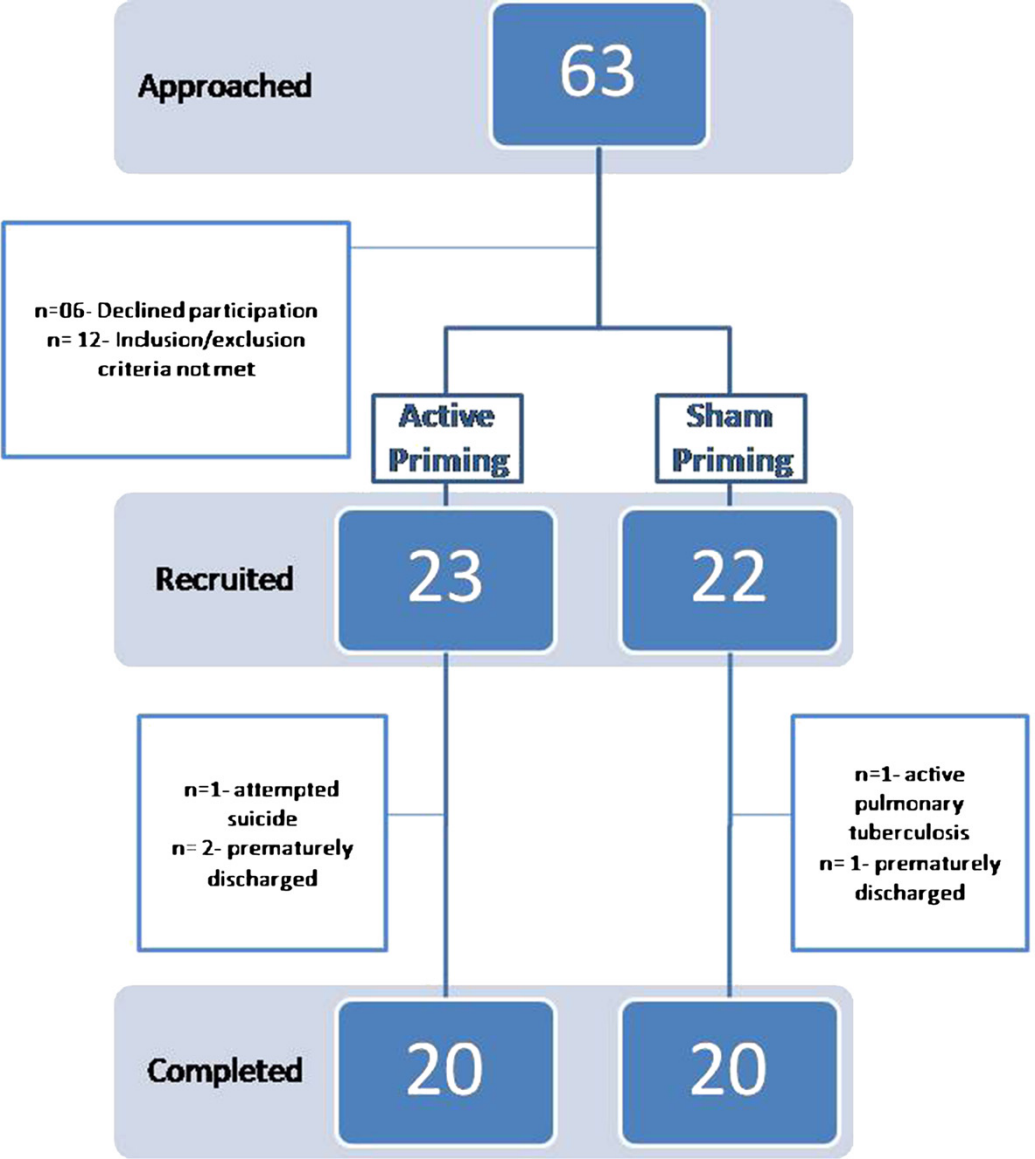
**Flow of subjects through the study.**

Patients met diagnostic criteria for schizophrenia (using the Diagnostic Criteria for Research (DCR) of International Classification of Diseases - tenth edition (ICD-10)) [12]. Duration of illness for inclusion was less than 2 years. An additional inclusion criterion was that the patients should not have been tried on more than one antipsychotic medication on an adequate dose, for an adequate duration. Exclusion criteria included a prior history of a seizure not induced by drug withdrawal, patients receiving ECT in the last 6 months, significant neurological illness or head trauma, significant unstable medical condition, presence of metallic implants, cardiac pacemakers etc., left-handedness, current drug abuse, or inability to provide informed consent. Age range was 18 to 60. Number of patients targeted for this study was 40. A total of 63 patients were screened. Six subjects did not agree to participate and 12 subjects did not meet inclusion/exclusion criteria: two patients had received ECT previously within the preceding 6 months, three had comorbid cannabis dependence syndrome, one had frequent exacerbations of asthma, two had frequent episodes of headache, one had hearing impairment, and three were hostile. A total of 45 patients were enrolled, out of which five patients who dropped out due to different reasons (one patient attempted suicide and sustained serious injuries, one was diagnosed with pulmonary tuberculosis and three were discharged before completion of study) (see Figure 1 for the flow diagram of the progress through the phases of a parallel randomized trial of two groups). All patients were inpatients and underwent regular physical examination, routine laboratory studies, and ECG. Patients were required to remain on their psychotropic medication at steady dosages for the duration of the trial.

The study protocol was approved by the institute ethics committee and a signed informed consent was obtained from every patient.

### TMS protocol

Patients (*n* = 40) were randomly allocated (using block randomization method) to active priming rTMS - ‘experimental group’ and sham priming rTMS - ‘control group.’ The decision to enroll a patient was always made prior to randomization. The two groups were comparable in terms of age, gender, occupation, education, marital status, drug status, past and family history, and indices of illness severity (Table 1). Patients were studied using a double-masked, parallel design, i.e., study participants and clinical raters remained masked to allocated condition and parameters.

**Table 1:** **Comparison of socio-demographic and clinical characteristics**

**Variable**		**Active group (** ***N*** **= 20) (mean** ± **SD)** **/** ***N*** **(%)**	**Sham group (** ***N*** **= 20) (mean** ± **SD)** **/** ***N*** **(%)**	***t*** **/** ***χ*** ^**2**^	**df**	***p***
Age in years		31.35 ± 7.13	29.30 ± 8.71	0.815	38	0.420
Education	Illiterate	4(20)	0(0)	4.224	2	0.167
	≤10th class	7(35)	8(40)			
	>10th class	9(45)	12(60)			
Occupation	Unemployed	5(25)	8(40)	1.026	1	0.311
	Working	15(75)	12(60)			
Marital status	Single	8(40)	10(50)	0.404	1	0.525
	Married	12(60)	10(50)			
Habitat	Rural	13(65)	18(90)	3.584	1	0.127
	Urban	7(35)	2(10)			
Family type	Nuclear	2(10)	4(20)	0.784	1	0.661
	Joint/extended	18(90)	16(80)			
Income (per month)	<3,000	8(40)	7(35)	0.477	2	0.788
	3,000–6,000	5(25)	7(35)			
	>6,000	7(35)	6(30)			
Diagnosis	F20.0	18(90)	14(70)	2.50	1	0.235
	Others	2(10)	6(30)			
Treatment (chlorpromazine equivalent/day)		499.77 ± 231.52	404.06 ± 181.39	1.455	38	0.154
Motor threshold	Priming	37.20 ± 7.78	38.25 ± 8.01	−0.421	38	0.676
	Stimulation (1-Hz stimulation)	41.50 ± 8.60	42.50 ± 10.21	−0.361	38	0.720

### TMS stimulation parameters

A Magstim Rapid® device (MAGSTIM Ltd., Whitland, Wales) and a figure-eight coil were utilized in the delivery of rTMS. Stimulation was administered at 100% resting motor threshold (RMT), which was ascertained prior to each stimulation session. Motor threshold was based on Rossini-Rothwell algorithm [13] defined as the lowest intensity, which produced five MEP responses of at least 50 μV in ten trials. Priming stimulation was provided over left temporo-parietal region (located according to the International 10-20 System of Electrode Placement’s PT3 location) 6 Hz, 20 trains of 5-s duration. For the control group same stimulation condition was used but with sham coil. Following priming or sham priming stimulation, both the groups received 1-Hz stimulation over the same site at 1 Hz continuous, 20-min train delivering 1,200 stimulations. Both groups received a total of 10 rTMS sessions (five days per week for 2 weeks).

### Clinical measures

Clinical measures used in this study were auditory hallucination subscale (AHRS) of the Psychotic Symptom Rating Scales (PSYRATS) [14]; composite positive and negative symptoms assessed using the Positive and Negative Syndrome Scale (PANSS) [15], and the Clinical Global Impression (CGI) [16] scores. Assessment with PSYRATS, PANSS, and CGI at baseline and after 1, 2, 4, and 6 weeks was done by an independent rater to ascertain double-blinding.

The primary outcome measure was AHRS scores at completion of ten sessions of rTMS, i.e., after 2 weeks. Scores at the end of 1 (suggesting early response), 4, and 6 weeks (suggesting maintenance of response) were secondary outcome measures. Scores on other measures too were secondary outcome measures.

### Statistical analysis

Study data were analyzed using SPSS (version 22). Statistical testing was two-sided with 5% as criterion for significance. Bonferroni correction for multiple comparisons was applied wherever applicable. The assumption of normality was verified by normal probability plots and Kolmogorov-Smirnov test. The main analysis was the effect of treatment over time and group interaction between the active and sham groups in the double-blind phase.

Group differences for sample characteristics as well as clinical parameters were examined with independent *t*-test and chi-square test (wherever applicable). Overall effect of treatment over time for the two groups was compared employing a set of multivariate repeated measures analysis of variance with treatment as the between-group factor and time as the within-subject factor. *Post hoc* tests were performed to look for differences across individual time points.

## Results

**Figure 2:**
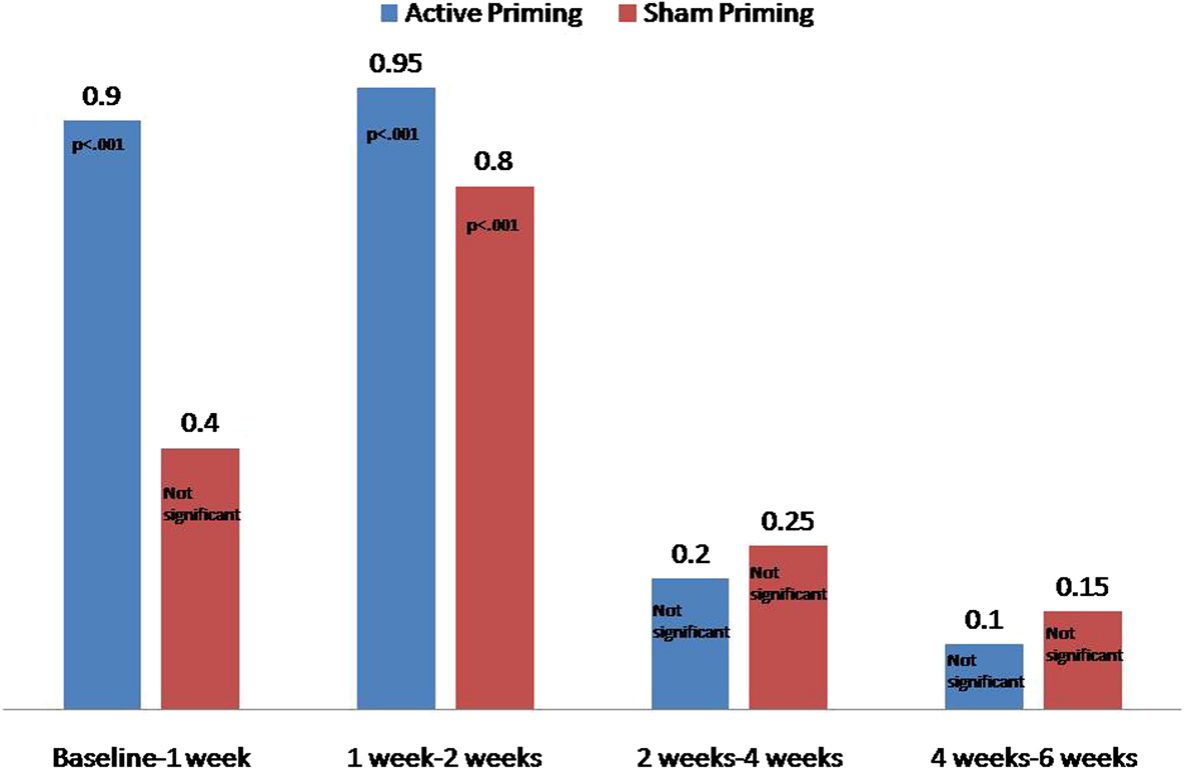
**Comparison of mean difference of change in loudness scores.**

On comparing the socio-demographic profile, no statistical significant difference could be found between active and control groups. The medication dosage and stimulation parameters were also comparable between the two groups (Table 1). Duration of illness was 4.85 ± 4.38 months (range: 1–19 months) in active priming group and 5.45 ± 4.54 (range: 1–22 months) in sham priming group (*p* = 0.673). None of the patients in active or sham group were drug naïve or drug free. The mean duration of treatment in active and sham group were 5.2 ± 5.17 (range: 1–18 weeks) and 4.7 ± 5.08 weeks (range: 1–19 weeks), respectively (*p* = 0.769). Table 2 shows comparison of mean scores and main effects of time, group, and time*group interaction within and between the two groups on PANSS, AHRS, and CGI subscales. We found that all the scores of these ratings significantly reduced over time (i.e., baseline through 6 weeks). These significant reductions persisted even after applying Bonferroni correction for multiple comparisons (i.e., *p* < .0125 for PANSS, *p* < .0042 for AHRS, and *p* < .0167 for CGI scores, respectively). However, except for the loudness subscale of AHRS, no other score showed significance between groups or time*group interaction. Loudness showed significant time*group interaction (*F* = 2.72; *p* < .05). Specifically, there was significant difference in the reduction in scores at first week compared to baseline. Active priming showed significant reduction in loudness at first week compared to baseline (*p* < .001), and sham priming did not result in significant change (Figure 2). Here, we also see that reduction through first week to sixth week in the two groups is statistically not significantly different.

**Table 2:** Main effects of time, group, and time*group interaction within and between the two groups on various outcome scales

**Variables**		**Active group (mean ± SD)**	**Sham group (mean ± SD)**	**Effect**	**Huynh Feldt** ***F***	***p***	**Partial eta squared**	**Observed power**
AHRS								
Frequency	Baseline	3.50 ± 0.89	2.80 ± 0.90	Time	*112.71*	*<0.001*	*0.75*	*1.00*
	1 week	2.35 ± 0.81	1.95 ± 1.10	Group	1.03	0.32	0.03	0.17
	2 weeks	1.05 ± 1.10	1.00 ± 1.10	Group*time	2.04	0.09	0.05	0.60
	4 weeks	0.75 ± 0.97	0.70 ± 0.86					
	6 weeks	0.80 ± 1.20	0.70 ± 0.92					
Duration	Baseline	2.75 ± 0.85	2.35 ± 0.75	Time	*116.02*	*<0.001*	*0.75*	*1.00*
	1 week	1.80 ± 0.83	1.45 ± 0.69	Group	1.22	0.28	0.03	0.19
	2 weeks	0.80 ± 0.77	0.70 ± 0.66	Group*time	0.89	0.44	0.02	0.28
	4 weeks	0.65 ± 0.88	0.60 ± 0.75					
	6 weeks	0.60 ± 0.99	0.40 ± 0.50					
Location	Baseline	4.00 ± 0.00	3.85 ± 0.73	Time	*40.08*	*<0.001*	*0.51*	*1.00*
	1 week	3.70 ± 0.73	3.45 ± 0.94	Group	0.41	0.50	0.01	0.10
	2 weeks	2.30 ± 1.95	2.20 ± 1.88	Group*time	0.10	0.93	0.003	0.07
	4 weeks	1.80 ± 2.04	1.45 ± 1.70					
	6 weeks	1.60 ± 2.01	1.25 ± 1.65					
Loudness	Baseline	2.60 ± 0.68	2.15 ± 0.75	Time	*111.28*	*<0.001*	*0.75*	*1.00*
	1 week	1.70 ± 0.66	1.35 ± 0.67	Group	0.55	0.46	0.01	0.14
	2 weeks	0.75 ± 0.72	0.85 ± 0.88	Group*time	*2.72*	*0.03*	*0.07*	*0.74*
	4 weeks	0.55 ± 0.69	0.60 ± 0.75					
	6 weeks	0.45 ± 0.60	0.45 ± 0.60					
Origin of voices	Baseline	4.00 ± 0.00	4.00 ± 0.00	Time	*40.59*	*<0.001*	*0.60*	*1.00*
	1 week	4.00 ± 0.00	3.75 ± 0.91	Group	0.01	0.91	0.00	0.05
	2 weeks	2.30 ± 1.95	2.20 ± 1.94	Group*time	0.15	0.88	0.004	0.07
	4 weeks	1.65 ± 1.93	1.80 ± 2.04					
	6 weeks	1.45 ± 1.88	1.45 ± 1.88					
Amount of negative content	Baseline	2.75 ± 0.77	2.50 ± 0.76	Time	*58.77*	*<0.001*	*0.61*	*1.00*
	1 week	2.25 ± 0.85	2.10 ± 0.91	Group	0.45	0.51	0.01	0.10
	2 weeks	1.30 ± 1.26	1.05 ± 1.15	Group*time	0.15	0.92	0.004	0.08
	4 weeks	0.95 ± 1.23	0.90 ± 1.21					
	6 weeks	0.80 ± 1.20	0.60 ± 0.99					
Degree of negative content	Baseline	2.65 ± 1.14	2.30 ± 1.03	Time	*36.28*	*<0.001*	*0.49*	*1.00*
	1 week	2.15 ± 1.04	1.65 ± 1.04	Group	0.80	0.38	0.02	0.14
	2 weeks	1.15 ± 1.31	1.10 ± 1.25	Group*time	0.47	0.68	0.01	0.14
	4 weeks	1.05 ± 1.47	0.80 ± 1.11					
	6 weeks	0.80 ± 1.32	0.60 ± 0.99					
Amount of distress	Baseline	2.95 ± 0.83	2.55 ± 0.76	Time	*78.57*	*<0.001*	*0.67*	*1.00*
	1 week	2.15 ± 0.99	1.75 ± 0.97	Group	0.73	0.40	0.02	0.13
	2 weeks	1.10 ± 1.25	1.00 ± 0.97	Group*time	0.72	0.55	0.02	0.20
	4 weeks	0.80 ± 1.11	0.70 ± 0.92					
	6 weeks	0.60 ± 0.99	0.55 ± 0.83					
Intensity of distress	Baseline	2.45 ± 0.95	2.20 ± 0.89	Time	*78.35*	*<0.001*	*0.67*	*1.00*
	1 week	1.75 ± 0.72	1.40 ± 0.82	Group	0.80	0.38	0.02	0.14
	2 weeks	0.85 ± 0.88	0.75 ± 0.71	Group*time	0.47	0.66	0.01	0.13
	4 weeks	0.70 ± 0.92	0.55 ± 0.69					
	6 weeks	0.50 ± 0.83	0.45 ± 0.60					
Disruption	Baseline	3.40 ± 0.88	0.88 ± 0.56	Time	*120.81*	*<0.001*	*0.76*	*1.00*
	1 week	2.55 ± 0.94	2.05 ± 0.76	Group	0.64	0.43	0.02	0.12
	2 weeks	1.20 ± 1.20	1.20 ± 1.15	Group*time	1.42	0.24	0.04	0.37
	4 weeks	0.90 ± 1.12	0.80 ± 1.05					
	6 weeks	0.65 ± 0.93	0.65 ± 0.93					
Control	Baseline	3.95 ± 0.22	4.00 ± 0.00	Time	*33.72*	*<0.001*	*0.47*	*1.00*
	1 week	3.95 ± 0.22	3.75 ± 0.91	Group	0.01	0.91	0.00	0.05
	2 weeks	2.40 ± 2.01	2.40 ± 2.01	Group*time	0.06	0.95	0.002	0.06
	4 weeks	1.80 ± 2.04	1.75 ± 2.00					
	6 weeks	1.60 ± 2.01	1.60 ± 2.01					
Total	Baseline	35.00 ± 5.34	31.70 ± 5.31	Time	*103.85*	*<0.001*	*0.73*	*1.00*
	1 week	28.35 ± 5.28	24.65 ± 7.81	Group	0.54	0.47	0.01	0.11
	2 weeks	15.20 ± 13.22	14.45 ± 12.73	Group*time	0.48	0.67	0.01	0.13
	4 weeks	11.60 ± 13.53	10.65 ± 12.30					
	6 weeks	8.25 ± 9.70	7.45 ± 9.69					
CGI								
Severity	Baseline	5.25 ± 0.97	5.15 ± 0.93	Time	*236.40*	*<0.001*	*0.86*	*1.00*
	1 week	4.00 ± 0.86	4.00 ± 0.86	Group	0.49	0.49	0.01	0.11
	2 weeks	2.75 ± 0.79	2.95 ± 0.69	Group*time	1.71	0.16	0.04	0.45
	4 weeks	2.25 ± 1.02	2.60 ± 0.99					
	6 weeks	1.90 ± 1.02	2.30 ± 0.80					
Improvement	Baseline	0.00 ± 0.00	0.00 ± 0.00	Time	*202.87*	*<0.001*	*0.84*	*1.00*
	1 week	2.60 ± 0.68	2.80 ± 0.52	Group	0.69	0.41	0.02	0.13
	2 weeks	1.90 ± 0.79	1.85 ± 0.59	Group*time	0.96	0.43	0.03	0.30
	4 weeks	1.65 ± 0.75	1.85 ± 0.67					
	6 weeks	1.35 ± 0.75	1.60 ± 0.60					
Efficacy	Baseline	0.00 ± 0.00	0.00 ± 0.00	Time	*127.29*	*<0.001*	*0.77*	*1.00*
	1 week	7.60 ± 1.96	8.15 ± 1.90	Group	0.63	0.43	0.02	0.12
	2 weeks	5.25 ± 2.53	5.20 ± 2.07	Group*time	0.62	0.62	0.02	0.18
	4 weeks	3.30 ± 2.43	4.20 ± 3.46					
	6 weeks	2.30 ± 2.25	2.90 ± 2.45					
PANSS								
PANSS-PS	Baseline	26.65 ± 5.72	28.20 ± 13.52	Time	*157.25*	*<0.001*	*0.81*	*1.00*
	1 week	19.25 ± 4.40	20.70 ± 7.81	Group	1.36	0.26	0.03	0.29
	2 weeks	13.85 ± 4.52	16.45 ± 6.91	Group*time	1.23	0.27	0.03	0.19
	4 weeks	11.45 ± 4.06	15.70 ± 12.43					
	6 weeks	10.55 ± 4.19	13.60 ± 8.11					
PANSS-NS	Baseline	23.15 ± 4.64	23.45 ± 5.03	Time	*200.78*	*<0.001*	*0.84*	*1.00*
	1 week	17.15 ± 4.59	18.60 ± 5.99	Group	0.79	0.47	0.02	0.20
	2 weeks	13.70 ± 4.88	15.55 ± 4.54	Group*time	0.74	0.40	0.02	0.13
	4 weeks	12.20 ± 4.23	13.40 ± 4.31					
	6 weeks	11.50 ± 3.52	12.45 ± 4.02					
PANSS-GP	Baseline	41.75 ± 7.92	39.25 ± 4.79	Time	*232.36*	*<0.001*	*0.86*	*1.00*
	1 week	31.95 ± 5.61	31.00 ± 3.73	Group	2.41	0.09	0.03	0.48
	2 weeks	25.70 ± 4.96	25.85 ± 2.96	Group*time	0.04	0.84	0.001	0.05
	4 weeks	22.95 ± 4.91	24.45 ± 4.58					
	6 weeks	21.75 ± 4.70	22.25 ± 4.19					
PANSS-total	Baseline	91.55 ± 14.71	87.75 ± 9.83	Time	*327.66*	*<0.001*	*0.90*	*1.00*
	1 week	68.70 ± 10.98	68.90 ± 9.98	Group	1.86	0.16	0.05	0.35
	2 weeks	53.25 ± 12.49	54.65 ± 9.07	Group*time	0.05	0.83	0.001	0.06
	4 weeks	46.60 ± 11.65	50.00 ± 11.79					
	6 weeks	43.95 ± 11.19	46.15 ± 11.54					

Values in italics indicate statistical significance.

## Discussion

To the knowledge of the authors, this is the first randomized sham-controlled trial comparing high-frequency priming of low-frequency rTMS for early phase treatment of AVH in recent onset schizophrenia subjects. In accordance with the existing evidence on the effect of low-frequency stimulation of left temporo-parietal site for treating AVH [4], our study groups (both) showed a significant reduction in the AVH scores (on all studied vectors). Interestingly, significant reduction over treatment sessions was found on overall positive, negative, and general psychopathology as well as clinical global improvement. Effect sizes, for our results varied from 0.47 (on control vector of AHRS) to 0.90 (PANSS total score), were comparatively larger than the earlier studies.

The two other trials [9,11] using priming rTMS for AVH found this paradigm did not lead to significant improvement over sham treatment; they found low-frequency stimulation alone also to be ineffective in treating AVH. Our study found active and sham priming of low-frequency rTMS did not show significant differences in reducing AVH. These earlier studies used 20 min of the total stimulation while we used 25 min of total stimulation; it took lesser number of total sessions (ten compared to 15 in earlier studies) for the current finding. Another important methodological difference was the intensity of stimulation. While Blumberger et al. [9] used 90% of RMT for priming and 115% for 1-Hz stimulation and Slotema et al. [11] used 80% of RMT for priming and 90% for 1-Hz stimulation, we used 100% RMT intensity for both stimulation frequencies.

In our study, it was observed that while both primed and sham primed low-frequency rTMS resulted in reduction of AVH, priming added an advantage in the reduction of loudness of the hallucinatory voices. High-frequency (6 Hz) stimulation delivered as priming might have lead to the additional benefit in the active group. However, studies comparing high- and low-frequency stimulations of temporo-parietal regions have found no significant differences (for review, see Slotema et al. [4]). For generalizing the specific benefits of theta range frequency, as used in this study, for high-frequency priming needs further evidence. Perhaps this benefit could be optimized in manipulating priming parameters further, e.g., the intensity and frequency of the priming stimulation. Patients with AVH might be facilitated with increased intensity (>100% RMT) or theta burst paradigms for priming stimulation in future studies. The study finding of reduction in AVH in schizophrenia with low-frequency magnetic stimulation is comparable to inhibitory stimulation delivered to the left temporo-parietal area with transcranial direct current stimulation (tDCS) [17]. However, more evidence for efficacy is available for rTMS than for tDCS, although there is a certain lack of head-to-head trials comparing tDCS and rTMS [18].

While loudness vector is a clearly defined sensory characteristic of AVH, it has been understood as one of the most salient perceptual characteristics from a perspective of phenomenology. It has been suggested to quantitatively distinguish AVH from intrusive thoughts and normal verbal thought [19]. Interestingly, loudness of AVH showed significant group difference, with early reduction in active priming group, earlier trials found no difference between the two forms of stimulation on any of the efficacy measures. Vercammen et al. [20] found that activation in temporo-parietal structures had a negative correlation with perceived loudness of AVH. While suggesting that louder AVH take up more resources involved in the processing of inner speech resulting in a reduction of task-related activity, they concluded that strong activation of the inner speech processing network may contribute to the subjective loudness of AVH. Our study findings imply that additional high-frequency priming of low-frequency stimulation of the left temporo-parietal site induce faster deactivation of inner speech network.

### Limitations

One major limitation of the study is the lack of sham stimulation control for low-frequency rTMS, which checked us from drawing conclusions on the efficacy of the stimulation paradigm. Moreover, limited sample size is a confound in generalizing the results.

## Conclusion

We conclude that both low-frequency rTMS alone and high-frequency priming of low-frequency rTMS are effective in treating overall psychopathology, particularly AVH when given in recent onset schizophrenia patients. Add on priming, however, seems to be particularly effective in faster reduction in loudness of AVH.
